# Haemolymph microbiome of the cultured spiny lobster *Panulirus ornatus* at different temperatures

**DOI:** 10.1038/s41598-019-39149-7

**Published:** 2019-02-08

**Authors:** Mei C. Ooi, Evan F. Goulden, Gregory G. Smith, Andrew R. Bridle

**Affiliations:** Institute for Marine and Antarctic Studies, University of Tasmania, Tasmania, Australia

## Abstract

Lobsters have an open circulatory system with haemolymph that contains microorganisms even in the healthy individuals. Understanding the role of these microorganisms becomes increasingly important particularly for the diagnosis of disease as the closed life-cycle aquaculture of the spiny lobster *Panulirus ornatus* nears commercial reality. This study aimed to characterise haemolymph responses of healthy cultured *P*. *ornatus* juveniles at control (28 °C) and elevated (34 °C) temperatures. This was assessed by measuring immune parameters (total granulocyte counts, total haemocyte counts, clotting times), and culture-independent (pyrosequencing of haemolymph DNA) and culture-dependent (isolation using nonselective growth medium) techniques to analyse bacterial communities from lobster haemolymph sampled on days 0, 4 and 6 post-exposure to the temperature regimes. Elevated temperature (34 °C) affected lobster survival, total granulocyte counts, and diversity, load and functional potential of the haemolymph bacterial community. Pyrosequencing analyses showed that the core haemolymph microbiome consisted of phyla Proteobacteria and Bacteriodetes. Overall, culture-independent methods captured a higher bacterial diversity and load when compared to culture-dependent methods, however members of the *Rhodobacteraceae* were strongly represented in both analyses. This is the first comprehensive study providing comparisons of haemolymph bacterial communities from healthy and thermally stressed cultured juvenile *P*. *ornatus* and has the potential to be used in health monitoring programs.

## Introduction

The ornate spiny lobster *Panulirus ornatus* is sought after as a high-end seafood product^1^. It has been produced by sea-cage aquaculture for more than 25 years in parts of south east Asia, where seedstock is sourced from the wild and grown to market size over an 18 month period^1^. However, since the late 1990 s the University of Tasmania has been developing closed life-cycle production technologies to breed and grow *P*. *ornatus* from egg to adult. This research has now spawned a company to commercialise the technology (UTAS Nexus Aquasciences - UNA), and is expected to significantly change the landscape of lobster industries by reducing wild seedstock harvesting and bolstering of seafood production and stock enhancement programs. The ongoing success of a commercialised closed life-cycle lobster sector will rely on better knowledge of cultured lobster health, with particular focus on non-lethal haemolymph sampling and microbiomic information that allows for health status assessment.

Environmental stressors, in particular temperature, can significantly impact animal health and productivity. For poikilothermic lobsters, ambient temperatures directly affect oxygen utilisation, metabolism, growth and moulting^2,3^, and elevated temperatures can drastically impact the lobster immune system^4^. Sea-cage grown *P*. *ornatus* is preferentially cultured in shallow coastal bays, where the animals are protected from strong wind or wave action, but the minimal water flushing in these areas subject animals to sporadic water temperatures^5^ well above optimal (25–31 °C). We explored the upper thermal limit of *P*. *ornatus* in a pilot study and found mortalities began at 35 °C after 3 days of exposure (unpub. data). This information becomes significant in the context of climate change, as elevated sea temperatures are becoming more frequent and have the potential to cause higher lobster mortality rates^6^.

Temperature stress may also significantly affect host-microbiota interactions in lobsters and other decapods, including those within the circulatory system^7,8^. Most invertebrates have open circulatory systems in which haemolymph (equivalent to blood) is actively circulated around the haemocoelic cavity^9^ and returns to the heart via the ostia^10^. Healthy invertebrates harbour a range of bacteria in their blood without exhibiting systemic disease and associated clinical signs^11,12^. This condition is termed asymptomatic bacteraemia^13^ and has been found so far in crustaceans^11^ and molluscs^12,14^. Bacteria in the haemolymph of crustaceans may positively impact host health by modulating the immune response, producing antimicrobial substances and competing with pathogens^11^. However, temperature stress may cause dysbiosis of haemolymph communities leading to proliferation and increased bacterial load (septicaemia), which has been reported in red swamp crawfish *Procambarus clarkii*^7^ and blue crab *Callinectes sapidus*^8^.

**Figure 1:**
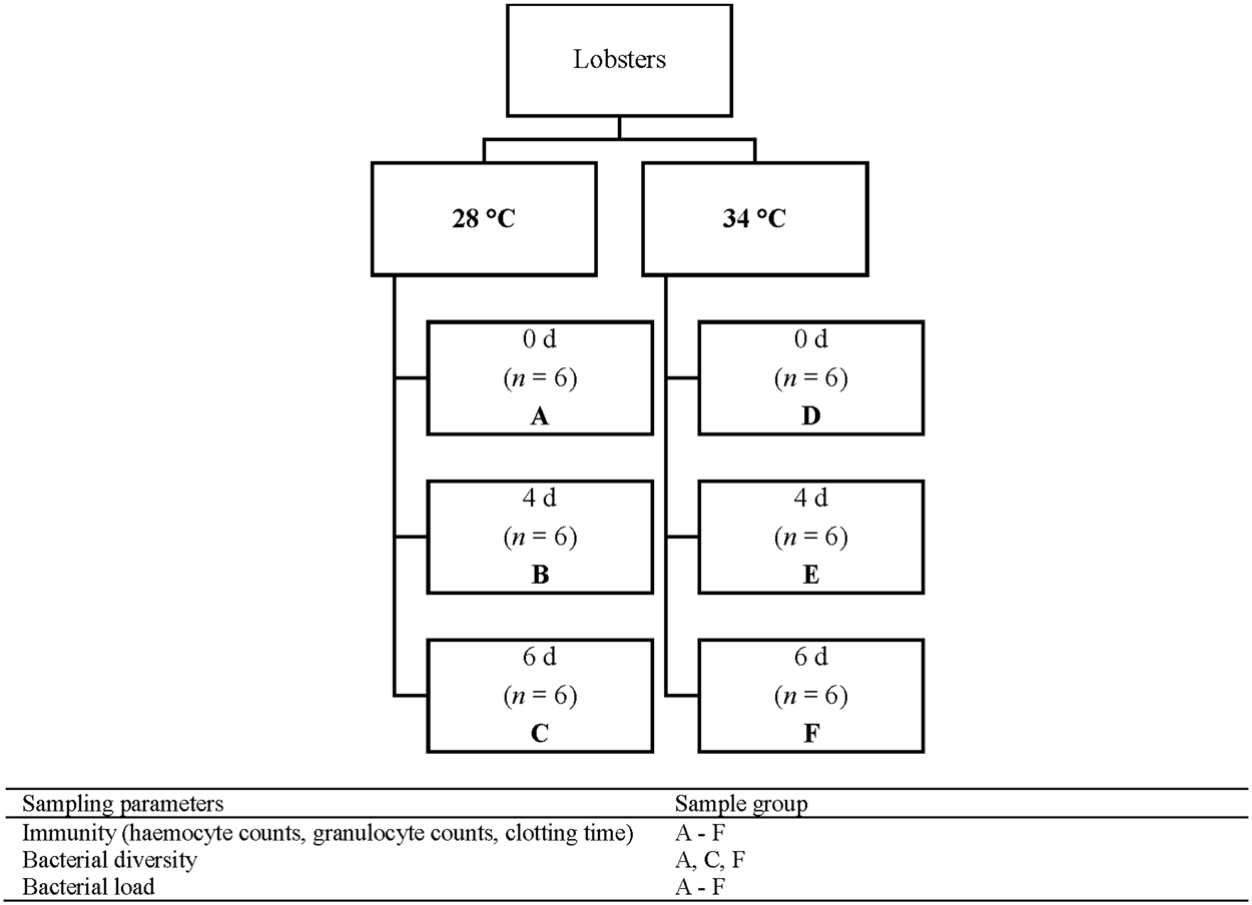
Experimental design showing sample groups and sampling parameters.

Previously, bacteria were found in the haemolymph of 5–100% of lobster species sampled by culture-dependent methods, including *P*. *ornatus*^15^, western rock lobster *P*. *cygnus*^16^, scalloped spiny lobster *P*. *homarus*^17^, southern rock lobster *Jasus edwardsii*^18^ and American lobster *Homarus americanus*^19^. However, recently there has been a shift to employ culture-independent techniques (e.g., targeted and non-targeted sequencing) that have increased sensitivity and that have been used to discover bacteria in the haemolymph in 100% of lobsters^20^. Although bacteria have been detected in the haemolymph of lobsters, limited information exists for the bacterial composition and diversity associated with healthy lobsters^20^, which makes the identification of potentially invasive and pathogenic microorganisms difficult. Furthermore, the effect of temperature on the bacterial communities and lobster immune responses is unknown. The present study aimed to characterise and quantify (1) haemolymph immune responses (e.g., total haemocyte and granulocyte counts, clotting times) and (2) haemolymph microbial communities (e.g., diversity, composition, core microbiome) using a combination of culture-dependent and culture-independent techniques (next generation sequencing, quantitative PCR) of juvenile *P*. *ornatus* exposed to optimal culture temperature (28 °C) and temperature stress (34 °C) just below the perceived thermal lethal limit (Fig. 1). Animals were exposed to 6 days of the thermal regimes to ensure a stress response but short enough to avoid mortality.

## Results

### Survival rate and immune parameters

**Figure 2:**
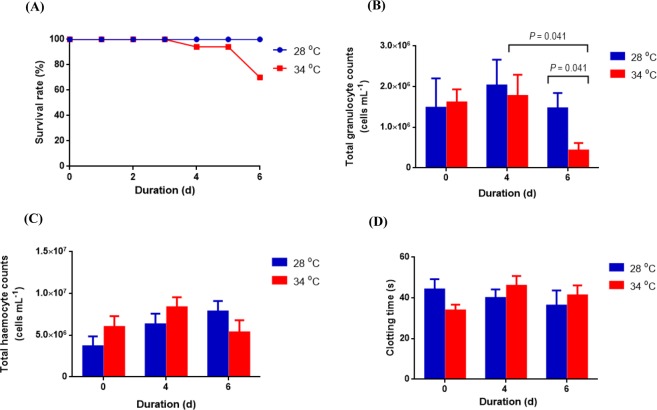
(**A**) Survival rate (%), (**B**) total granulocyte counts (cells mL^−1^), (**C**) total haemocyte counts (cells mL^−1^) and (**D**) clotting time (s) of *P*. *ornatus* juveniles exposed to 28 °C or 34 °C. Each bar represents mean + SEM, *n* = 6 (except clotting time on days 4 and 6 with *n* = 4).

Survival and immune parameters were measured to assess the impact of temperature on *P*. *ornatus*. There was no mortality until the fourth day of exposure to 34 °C; the survival rates were 94% on 4 days post-exposure (dpe) and 70% on 6 dpe (Fig. 2A). No mortality was observed in the 28 °C treatment. The total granulocyte counts of lobsters exposed to 34 °C on 6 dpe dropped significantly when compared to those on 4 dpe (U = 5.000, *P* = 0.041) and control animals on 6 dpe (U = 5.000, *P* = 0.041) (Fig. 2B). Total haemocyte counts ranged from 4.8 × 10^5^–1.3 × 10^7^ cells mL^−1^, however no significant difference (*P* > 0.05) was found between animals exposed to 34 °C and 28 °C (Fig. 2C). Clotting times did not significantly differ (*P* > 0.05) between juveniles held at either temperature and ranged between 18–57 s (Fig. 2D).

### Bacterial diversity

#### Summary of pyrosequencing

V1 to V3 hypervariable regions of the bacterial 16S rRNA gene in the haemolymph sample libraries were sequenced. A total of 104,183 reads from 20 samples were obtained after quality filtering and removal of chimeric sequences (Table 1). Mean reads per sample was 5,209 and mean read length was 302 bp. Good’s coverage ranged from 93.3 to 98.6% (Table 1).

**Table 1:** Sampling depth, richness and alpha diversity indices for haemolymph sequence libraries of juvenile *P*. *ornatus*.

Sample ID	Lobster no.	Sampling depth									Richness estimators		Diversity indices	
		Raw sequences	Filtered sequences	Obs. OTUs	Good’s coverage (%)	Phylum	Class	Order	Family	Genus	Chao1	ACE	Shannon	Simpson
**28 °C day 0**														
**Culture-independent samples**														
28C0d1	1	7,532	4,354	109	97.5	6	11	19	18	21	144	145	2.54	0.81
28C0d2	2	4,790	2,660	119	95.5	7	10	16	16	15	130	136	3.71	0.96
28C0d3	3	4,248	2,389	160	93.3	6	12	16	14	18	177	181	4.39	0.98
28C0d4	4	9,229	6,979	230	96.7	8	13	25	30	32	320	280	4.19	0.95
28C0d5	5	9,689	6,978	274	96.1	11	19	32	35	39	344	367	4.33	0.96
28C0d6	6	4,972	3,755	237	93.7	13	19	31	30	34	281	318	4.26	0.97
**28 °C day 6**														
**Culture-independent samples**														
28C6d1	1	5,132	3,745	172	95.4	7	11	22	24	21	198	204	3.76	0.93
28C6d2	2	8,588	6,261	137	97.8	5	9	13	14	16	154	158	4.16	0.98
28C6d3	3	8,911	5,439	158	97.1	6	9	15	16	11	206	197	4.11	0.97
28C6d4	4	4,264	3,149	156	95.0	8	10	20	21	27	176	179	4.15	0.97
28C6d5	5	4,869	2,325	145	93.8	9	12	16	22	29	161	158	4.08	0.97
28C6d6	6	9,043	6,169	108	98.2	4	7	13	15	19	145	138	3.61	0.95
**Culture-dependent samples**														
28C6d128^a^	1–6	12,306	10,051	140	98.6	2	3	6	4	11	155	159	2.56	0.85
**34 °C day 6**														
**Culture-independent samples**														
34C6d1	1	3,810	2,678	108	96.0	3	6	11	13	18	121	128	3.62	0.96
34C6d2	2	10,382	8,576	156	98.2	5	11	20	16	26	225	193	3.92	0.96
34C6d3	3	10,046	5,464	77	98.6	5	9	15	18	21	104	107	1.73	0.61
34C6d4	4	9,007	5,516	112	98.0	5	9	13	14	7	144	136	3.71	0.95
34C6d5	5	8,872	5,524	112	98.0	6	9	12	11	10	132	137	3.58	0.95
34C6d6	6	5,092	3,107	133	95.7	7	11	20	22	23	151	151	3.84	0.96
**Culture-dependent samples**														
34C6d178^a^	1–6	12,012	9,064	197	97.8	2	4	6	7	14	203	205	3.66	0.94

^a^Pool of all colonies cultured on marine agar.

#### Alpha diversity analyses

Rarefaction curves are in Supplementary Fig. 1. The dataset was subsampled to a depth of 2,325–10,051 (Table 1). The observed operational taxonomic units (OTUs) for the haemolymph of control lobsters (28 °C) ranged from 108 to 172 on 6 dpe (Table 1). The haemolymph of animals exposed to 34 °C for 6 d had 77–156 observed OTUs. There were no significant differences in the observed OTUs (*t*_10_ = 2.106, *P* = 0.061), richness estimators (Chao1 [*t*_10_ = 1.366, *P* = 0.202], ACE [*t*_10_ = 1.930, *P* = 0.082]), or diversity indices (Shannon [U = 6.000, *P* = 0.065], Simpson [U = 9.000, *P* = 0.158) between juveniles held at either temperature for 6 d (Table 1).

#### Beta diversity analyses

**Figure 3:**
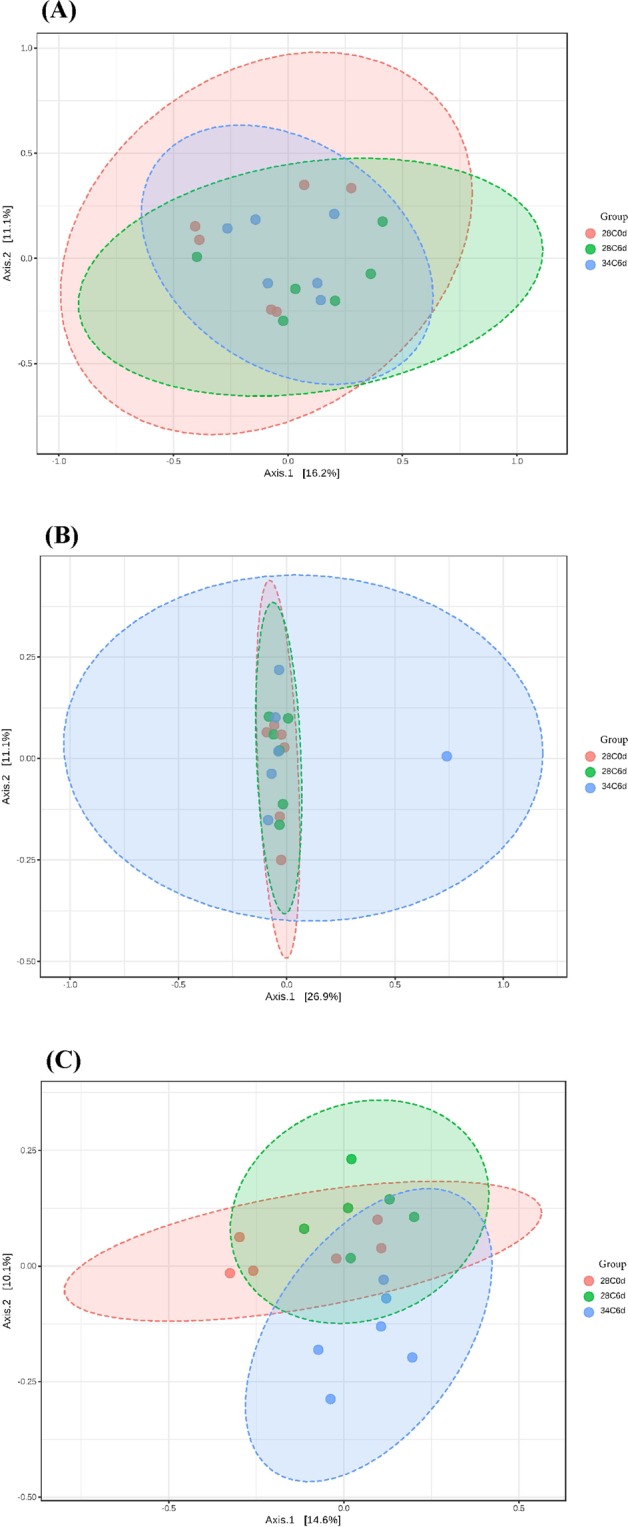
Principal coordinate analysis plots based on (**A**) Bray Curtis index, (**B**) weighted UniFrac and (**C**) unweighted UniFrac distance methods showing similarity in haemolymph sequence libraries of *P*. *ornatus* juveniles. Key: 28C0d: 28 °C 0 dpe; 28C6d: 28 °C 6 dpe; 34C6d: 34 °C 6 dpe.

The first two axes of principal coordinate analyses (PCoA) based on Bray Curtis, weighted UniFrac and unweighted UniFrac distance matrices explained 27.3, 38.0 and 24.7% of the variation in abundance of OTUs among different sample libraries, respectively (Fig. 3). This variation was not related to treatment groups except for the unweighted UniFrac where there was some separation along the second axis. The PCoA results were supported by PERMANOVA. When the Bray Curtis index (R^2^ = 0.143, *P* = 0.083), weighted UniFrac (R^2^ = 0.123, *P* = 0.357) and unweighted UniFrac (R^2^ = 0.150, *P* = 0.031) distance matrices were analysed statistically using PERMANOVA, only the last showed significant difference among the three types of culture-independent haemolymph libraries.

#### Taxonomic composition

**Figure 4:**
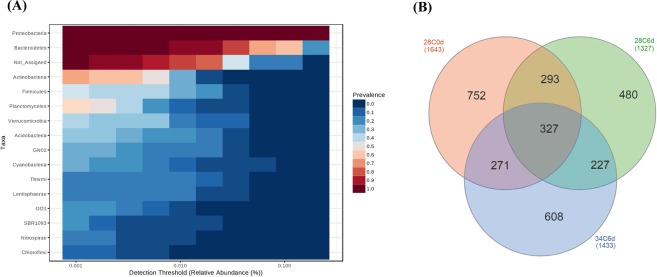
(**A**) Core microbiome analysis based on relative abundance and sample prevalence of bacterial OTUs grouped by phyla from haemolymph samples of juvenile *P*. *ornatus*. (**B**) Venn diagram showing shared and unique OTUs in haemolymph samples of juvenile *P*. *ornatus*. Key: 28C0d: 28 °C 0 dpe; 28C6d: 28 °C 6 dpe; 34C6d: 34 °C 6 dpe.

Culture-independent samples: Sequencing analysis of culture-independent haemolymph sample libraries indicated that the core microbiome of juvenile *P*. *ornatus* consisted of Proteobacteria and Bacteroidetes (Fig. 4A). This was based on highly abundant and prevalent OTUs grouped by phylum and excluded bacterial taxa not assigned (i.e. OTUs with 97% nucleotide sequence identity that were unable to be assigned to known bacterial taxa in the Greengenes database with a confidence threshold of 0.8 using RDP Bayesian Classifier). The Venn diagram (Fig. 4B) showed that the three groups of haemolymph samples shared 327 OTUs (11% of total OTUs) which included genera *Ruegeria*, *Acinetobacter*, *Pseudomonas*, *Loktanella*, *Cohaesibacter*, *Psychrobacter*, BD2_13, *Nautella*, *Phaeobacter*, *Thalassobius* and *Antarcticimonas*. The haemolymph of control lobsters on 6 dpe shared significantly more OTUs (χ^2^ = 8.377, *P* = 0.004) with those on 0 dpe (293 OTUs; 22% of 28C6d) than thermally challenged juveniles on 6 dpe (227 OTUs; 17% of 28C6d). Control animals on 0 dpe had the highest proportion of unique OTUs (752; 46% of 28C0d).

**Figure 5:**
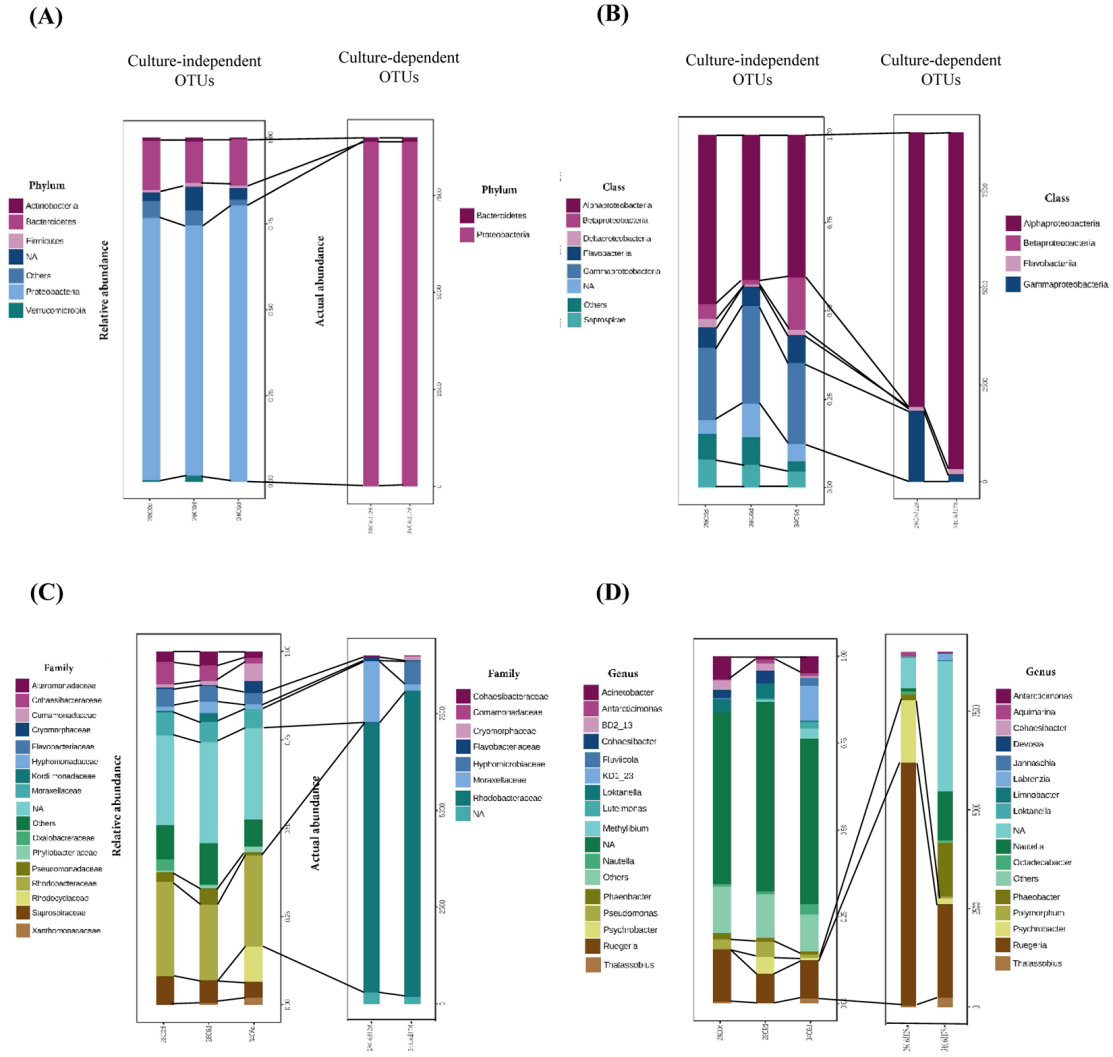
Comparisons of relative abundance (culture-independent) and actual abundance (culture-dependent) of OTUs in haemolymph sample libraries of juvenile *P*. *ornatus* at (**A**) phylum, (**B**) class, (**C**) family and (**D**) genus levels. Key: 28C0d: 28 °C 0 dpe; 28C6d: 28 °C 6 dpe; 34C6d: 34 °C 6 dpe; 28C6d128: cultured colonies from 28 °C 6 dpe; 34C6d178: cultured colonies from 34 °C 6 dpe; NA: not assigned to bacterial taxa based on classification methods used in this study.

The composition of haemolymph bacterial communities of control and stressed animals at day 6 were analysed using the MetagenomeSeq statistical pipeline at phylum, class, family and genus levels. The culture-independent haemolymph libraries of lobster groups (28 °C 0 dpe, 28 °C 6 dpe, 34 °C 6 dpe) were mostly dominated by Proteobacteria and Bacteroidetes (Fig. 5A). Phyla Verrucomicrobia (*P* = 0.046) and Thermi (*P* = 0.018) were significantly more represented in the haemolymph of control lobsters (Verrucomicrobia 1.7 ± 0.9%, Thermi 0.7 ± 0.5%) than that of thermally stressed animals on 6 dpe. The four major classes represented in the haemolymph libraries of juvenile lobsters were Alphaproteobacteria, Gammaproteobacteria, Saprospirae and Flavobacteriia (Fig. 5B). The haemolymph of animals exposed to 28 °C had significantly higher abundance of classes Verrucomicrobiae (1.6 ± 1.0%; *P* < 0.001), Deinococci (0.7 ± 0.5%; *P* < 0.001), Acidimicrobiia (0.4 ± 0.3%; *P* = 0.003), Clostridia (0.9 ± 0.6%; *P* = 0.024) and Lentisphaeria (1.0 ± 0.7%; *P* = 0.032) than that of juveniles exposed to 34 °C on 6 dpe. The most abundant bacterial families found in the haemolymph were *Rhodobacteraceae*, *Saprospiraceae*, *Flavobacteriaceae* and *Cohaesibacteraceae* (Fig. 5C). Families *Trueperaceae* (0.7 ± 0.5%; *P* = 0.020), *Kordiimonadaceae* (2.5 ± 1.7%; *P* = 0.039), *Bradyrhizobiaceae* (0.7 ± 0.5%; *P* = 0.043) and *Acidaminobacteraceae* (0.9 ± 0.6%; *P* = 0.045) were significantly more represented in the haemolymph of control juveniles compared to that of thermally stressed lobsters on 6 dpe. The predominating bacterial genera were *Ruegeria*, *Acinetobacter*, KD1_23, *Pseudomonas* and *Loktanella* (Fig. 5D). On 6 dpe, the haemolymph of 28 °C exposed juveniles had significantly higher abundance of *Loktanella* (4.2 ± 3.2%; *P* = 0.005), *Cohaesibacter* (3.8 ± 2.4%; *P* = 0.006), *Polaribacter* (0.6 ± 0.3%; *P* = 0.028) and *Micrococcus* (0.2 ± 0.2%; *P* = 0.028) than 34 °C exposed lobsters. Overall, the abundance of rare haemolymph OTUs at all taxonomic levels decreased in thermally stressed animals.

Culture-dependent samples: Haemolymph sequence libraries comprising pooled isolates cultured from lobsters either exposed to control or elevated temperature at day 6 were represented only by phyla Proteobacteria and Bacteroidetes (Fig. 5A). The two predominating classes of culturable bacteria belonged to the Alphaproteobacteria and Gammaproteobacteria (Fig. 5B). There were fewer families represented in the haemolymph libraries of culturable bacteria when compared to culture-independent bacteria, with the former dominated by *Rhodobacteraceae* and *Moraxellaceae* (Fig. 5C). The most represented culturable bacterial genera in the haemolymph samples were *Ruegeria*, *Psychrobacter*, *Phaeobacter* and *Nautella* (Fig. 5D). Genera *Acinetobacter*, *Pseudomonas* and BD2_13 were not represented in culture-dependent samples but featured in culture-independent sample libraries (Fig. 5D).

#### Functional potential

**Figure 6:**
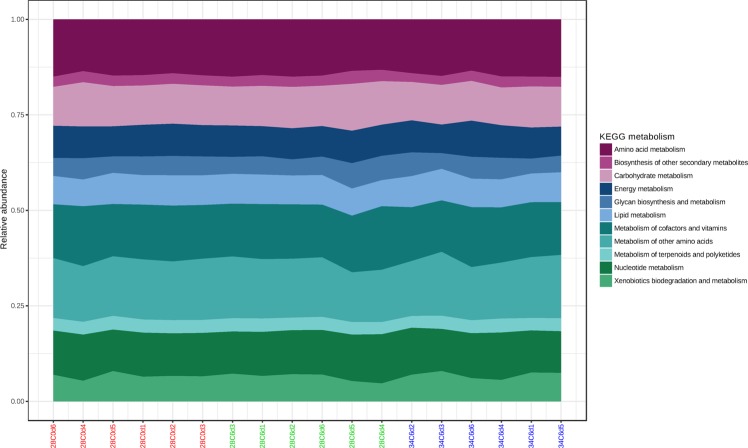
Functional diversity profiling of culture-independent OTUs in haemolymph samples of juvenile *P*. *ornatus* based on KEGG metabolism using PICRUSt. Key: 28C0d: 28 °C 0 dpe; 28C6d: 28 °C 6 dpe; 34C6d: 34 °C 6 dpe.

PICRUSt was employed to computationally predict gene families found in lobster haemolymph communities and metabolic functional profiles were then assigned using KEGG. Amino acid metabolism, metabolism of other amino acids and metabolism of cofactors and vitamins were identified as the main metabolic functions of lobster haemolymph communities (Fig. 6). Three KEGG pathways were significantly over represented in the haemolymph of control lobsters when compared to animals exposed to 34 °C on 6 dpe: sphingolipid metabolism (*P* = 0.020; K00720, K01201, K01190; lipid metabolism), phosphonate and phosphinate metabolism (*P* = 0.031; K01841, K05306; metabolism of other amino acids) and other glycan degradation (*P* = 0.044; K01190, K12111, K12112, K12373, K01201; glycan biosynthesis and metabolism).

### Bacterial load

**Figure 7:**
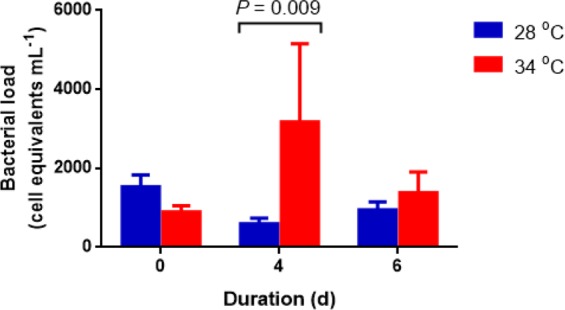
Bacterial load in the haemolymph of *P*. *ornatus* juveniles exposed to 28 °C and 34 °C based on culture-independent (*rpoB* gene) technique. Each bar is mean + SEM, *n* = 6.

The concentrations of bacteria among lobsters ranged from 0 to 3.19 × 10^3^ CFU mL^−1^ (culturable bacteria) and from 55 to 1.31 × 10^4^ cell equivalents mL^−1^ (*rpoB* gene, qPCR). The only significant difference in the bacterial load (U = 2.000, *P* = 0.009) was between the thermally stressed lobsters and control juveniles on 4 dpe (Fig. 7) as revealed by qPCR. There was no significant difference (*P* > 0.05) in the culturable bacterial load between animals exposed to 34 °C and 28 °C through time.

## Discussion

### Overview

This is the first study to comprehensively characterise and quantify the haemolymph microbiome of juvenile *P*. *ornatus*. We found that exposure to increased temperature reduced survival rate and affected haemolymph immune response (total granulocyte counts), the diversity, load (culture-independent method) and functional profile of haemolymph bacterial communities. Culture-independent bacterial community analyses captured a higher bacterial diversity and load when compared to the culture-dependent method, however both indicated phyla Proteobacteria and Bacteroidetes were predominating members of the haemolymph microbiome. We also showed that bacterial load of the haemolymph was understated using culture based plating methods when compared to qPCR, consistent with findings of those of Givens, *et al*.^21^ in analysis of haemolymph from crab *C*. *sapidus*. Our observed changes to haemolymph bacterial load and diversity of *P*. *ornatus* are consistent with other temperature stress studies on aquatic invertebrates including the Pacific oyster *Crassostrea gigas*^14^, crawfish *P*. *clarkii*^7^ and crab *C*. *sapidus*^8^.

### Survival and immune parameters

In the present study, lobster mortality occurred after 4 days of increased temperature, and was associated with signs of lethargy and opaque muscle. As optimal growth temperatures established for *P*. *ornatus* are in the range of 25 to 31 °C^22^, normal metabolism becomes compromised at higher temperatures and exposure to 34 °C may be approaching the physiological thermal limit. These observations were concomitant with a significant decrease in total granulocyte counts, which could have decreased the capacity to protect against opportunistic bacteria and leave the animal susceptible to infection^23^. Granulocytes are responsible for secreting phenoloxidase^24^, which activates the encapsulation of pathogens and tissue repair mechanisms (melanogenesis) in invertebrates. Phenoloxidase activity was found to decrease in response to increased temperatures in Pacific white shrimp *Litopenaeus vannamei*^25^ and yellowleg shrimp *Penaeus californiensis*^26^. Unlike total granulocyte counts, total haemocyte counts (THC) did not differ significantly between control and 34 °C animals in the present study. Studies have shown that acute thermal stress (<7 dpe) resulted in significant decreases in THC of Mediterranean shore crab *Carcinus aestuarii*^27^ and shrimp *L*. *vannamei*^25^, while chronic thermal stress (>14 dpe) can increase THC in lobster *H*. *americanus*^28^.

The thermal increment of 6 °C greater than the best practice culture temperatures did not have a profound effect on THC or clotting times in the haemolymph of *P*. *ornatus*. This could be due to the acute exposure time-frame used in the experiment (6 d). Previous studies have shown that changes to THC of thermally stressed lobster *H*. *americanus*^28^ and crab *C*. *aestuarii*^27^ were not observed until 7 dpe. Other possible reasons for the apparent lack of temperature effect on these parameters are the tight modulation of haemolymph by the host and associated microbiota. For example, cellular (i.e. haemocyte proliferation) and immune (i.e. increased phenoloxidase activity) parameters in the haemolymph of crab *C*. *aestuarii* were host regulated with temperature changes^27^.

### Bacterial diversity

The core microbiome of juvenile *P*. *ornatus* haemolymph consisted of Proteobacteria (Alpha-, Gamma-) and Bacteroidetes irrespective of thermal exposure treatment. Similarly, the majority of the bacteria cultured from the haemolymph of apparently healthy lobster species including *P*. *ornatus*^15^, *P*. *cygnus*^16^, *P*. *homarus*^17^, *J*. *edwardsii*^18^ and *H*. *americanus*^19^ belonged to Proteobacteria. Proteobacteria and/or Bacteroidetes were also the dominant phyla of bacterial communities in the circulatory system of other aquatic invertebrates, such as bivalve *C*. *gigas*^14^ and crab *C*. *sapidus*^21^. This may suggest the importance of these phyla in the host or reflect their ubiquitous distribution in the environment. Furthermore, as bacteria may be transmitted from the digestive tract to the circulatory system, the gut microbiome may be related to the haemolymph microbiome^29^. Our research group has recently shown that the core gut microbiome of juvenile *P*. *ornatus* cultured from the same facility shared Proteobacteria with the core haemolymph microbiome in addition to Tenericutes^30^. Bacterial families that were found in both the gut and haemolymph include *Flavobacteriaceae*, *Saprospiraceae*, *Rhodobacteraceae*, and *Moraxellaceae*.

*Rhodobacteraceae* were detected in all culture-dependent and culture-independent samples. *Rhodobacteraceae*, and in particular the genus *Ruegeria*, was predominant in an earlier culture-based study on bacteria in the haemolymph of *P*. *ornatus* juveniles from the same facility (unpub. data). *Ruegeria* commonly form symbiotic relationships with aquatic animals by producing antimicrobials and are major contributors of carbon and sulphur cycles in the marine environment^31–34^. In contrast, other members of *Rhodobacteraceae* such as *Nautella* also detected across haemolymph libraries in our study, are known marine pathogens implicated in algal bleaching^35^. Furthermore, Stratil, *et al*.^36^ reported an increase in *Rhodobacteraceae* abundance in brown macroalga *Fucus vesiculosus* with increasing temperature. Conversely, in our study the abundance of a haemolymph-associated *Rhodobacteraceae* genus (i.e. *Loktanella*) decreased under thermal stress.

Bacterial taxa that were characterised consistently by culture-dependent and culture-independent methods across all the experimental treatments in this study are likely to have important haemolymph functions or associations with *P*. *ornatus*. The persistence of *Cohaesibacteraceae*, *Flavobacteriaceae*, and *Moraxellaceae* in the haemolymph across all treatments suggests that these genera are resident members (commensals or symbionts) as opposed to transient populations^37^ of the haemolymph microbiome. Members of *Cohaesibacteraceae* including *Cohaesibacter* are known nitrate reducers, capable of converting nitrate to nitrite that may then be utilised by host nitric oxide synthases to generate nitric oxide^38–40^. This could be important for the immune response of lobsters as nitric oxide is involved in the antimicrobial defences of crustaceans^41,42^. *Flavobacteriaceae* have been reported to have symbiotic relationships with insects and produce enzymes that can degrade organic compounds^43^. *Psychrobacter* of family *Moraxellaceae* have been described to have antagonistic effects against pathogenic bacteria^44,45^, lipolytic activity and the ability to reduce nitrate to nitrite^46^. The persistence of *Psychrobacter* has been reported previously in the haemolymph of Dungeness crab *Cancer magister*^47^. On the other hand, Chistoserdov, *et al*.^48^ and Baross, *et al*.^49^ have linked the presence of *Flavobacteriaceae* and *Moraxellaceae* with disease in crustaceans, although there was no suggestion this was the case in our study.

The changes in haemolymph bacterial abundance from lobsters sampled on 6 dpe could indicate thermal stress had a dysbiotic effect on the communities. The majority of haemolymph OTUs impacted by elevated temperature have been reported in the marine environment such as *Kordiimonadaceae*^50^, *Acidaminobacteraceae*^51^, *Loktanella*^52^, and *Polaribacter*^53^. *Micrococcus* are known symbionts in marine sponges^54^. In most cases the relative abundance of the aforementioned lobster haemolymph OTUs were reduced or had disappeared at elevated temperature, which could infer significant function loss. Further investigation of these bacterial groups would be required as their decline or absence could indicate stress.

The predicted functions of bacterial communities generated by the PICRUSt algorithm may assist in explaining the role of bacteria in the haemolymph. In the present study, the main putative functions of haemolymph communities were metabolism of amino acid and other amino acids, which may be associated with amino acids and non-protein amino acids in the haemolymph^55^. Amino acid metabolism is also a major function of the global ocean microbiome^56^, which implies that bacterial-produced amino acids are crucial to oceanic trophic interactions and survival of host organisms. Phosphonate and phosphinate metabolisms were also affected by temperature, similar to the results of a salinity stress study on shrimp *L*. *vannamei*^57^. Phosphonate and phosphinate metabolism can only be conducted by protists and bacteria, particularly Actinobacteria^58^ which were found in *P*. *ornatus* haemolymph in this study. The third most abundant metabolic function involved vitamins and cofactors, which may not be synthesised or synthesised in limited quantities by lobsters^59^ rendering them dependent on bacteria in the haemolymph. Other KEGG pathways that were influenced by elevated temperature were glycan degradation (other) and sphingolipid metabolism. Both pathways are part of glycosphingolipid biosynthesis^58^. Sphingolipids in some bacteria and fungi may be involved in attachment to host or microbial cells^60^, and signalling during heat stress^61^. Functional changes of indigenous bacterial communities could be a driver in reducing host health in the present study, where specific bacterial metabolic pathways are diminished under certain conditions such as thermal stress.

### Implications

Non-lethal sampling of haemolymph is an important consideration when assessing asymptomatic bacteraemia in both healthy and diseased animals for effective health evaluations. It has been estimated for spiny lobsters (e.g., *Panulirus interruptus*) that haemolymph constitutes about 30% of the total animal wet weight^62^. Radford, *et al*.^63^ demonstrated previously that lobster *J*. *edwardsii* juveniles make a full recovery within 3 weeks following removal of 10–15% of haemolymph volume. Moreover, several studies that conducted repeat sampling from individual crustaceans^23,47,63–66^ and Evans^23^ found no decrease in protein level. In the present study, lobsters were euthanised due to the large volume of haemolymph required for several immune and bacterial assays. Only half a millilitre of haemolymph was used for culture-independent bacterial analyses, representing ~ 1% of the total haemolymph volume of the average weight of juveniles. Non-lethal sampling of such volumes of haemolymph would allow for animal recovery and provide a feasible means to conduct ongoing lobster health monitoring programs.

### Limitations

In this study, all cultured haemolymph bacteria were cultivated at 28 °C despite some communities being derived from lobsters exposed to 34 °C. We recognise this may have introduced a selective force bias, however this could have been offset by the growth of bacteria at 28 °C that would otherwise be in stressed or viable but non-culturable states when cultivated at 34 °C. This is supported in part by our existing analyses that still revealed differences in bacterial diversity between the culturable haemolymph communities of lobsters exposed to 28 °C and 34 °C although this could not be statistically validated due to the small (pooled) sample size. The nested PCR approach used for the culture-independent analyses of our samples may have also produced confounding results. It is generally accepted that next-generation amplicon sequencing read abundances are biased to varying degrees by 16S copy number, PCR primer mismatches and the number of PCR cycles^67^. 16S amplicon abundance bias remains unavoidable and as such we minimised the number of PCR cycles and therefore the potential for bias in our nested PCR. This approach was suggested previously by Yu *et al*.^68^ who proposed that nested PCR is acceptable when standard PCR cannot be used. Although the functional diversity profile of OTUs may be affected by the variation of 16S rRNA copy numbers in different bacteria^69^, the functional association analysis was compared between bacteria in the stressed and control lobsters which should have limited the copy number variations.

## Conclusion

This is the first study to characterise and quantify haemolymph responses and the microbiome of *P*. *ornatus* exposed to thermal challenge. Increased temperature (34 °C) affected survival, total granulocyte counts, bacterial diversity, bacterial load and changed the predicted functional profile of haemolymph bacterial communities. The core microbiome of the haemolymph as revealed by both culture-dependent and culture-independent analyses was represented by Proteobacteria (Alpha-, Gamma-) and Bacteroidetes. *Rhodobacteraceae* (Alphaproteobacteria) were prevalent across all samples. This study shows that non-lethal sampling and the subsequent examination of changes to the haemolymph microbiome has potential for health monitoring programs for spiny lobsters.

## Methods

### Experimental design

Juvenile *P*. *ornatus* were reared from hatch at the University of Tasmania’s Institute for Marine and Antarctic Studies (IMAS) located in Hobart, Australia. Culture methods were based on modified protocols from Jensen, *et al*.^70^ and Fitzgibbon and Battaglene^71^. Juveniles were cultured communally in 4,000 L fiberglass tanks within a recirculating system. Water quality parameters during culture were; temperature 28.1 ± 0.5 °C, pH 8.05 ± 0.1, salinity 34 ± 0.5 ppt and dissolved oxygen 95 ± 5%. Juveniles were fed once daily fresh blue mussel (*Mytilus galloprovincialis*) or squid (*Nototodarus gouldi*) at a rate of approximately 10% wet weight of total tank biomass. For this experiment, 44 lobsters (162.7 ± 4.6 g) were placed individually in oyster mesh cages and distributed in 6 × 600 L fiberglass tanks (7–8 lobsters per tank) where they were acclimated for 24 h in flow-through sea water (temperature 28.3 ± 0.03 °C; dissolved oxygen 92.8 ± 1.1%). The experiment started after this acclimation period. In three of the tanks, lobsters were exposed to thermal stress by increasing the temperature from 28 to 34 °C (33.9 ± 0.02 °C) over 28 h (Fig. 1). The remaining three tanks (control juveniles) were maintained at 28 °C (28.0 ± 0.02 °C). Animals were not fed during the six-day experiment.

#### Haemolymph sampling

On 0, 4 and 6 dpe, six randomly chosen lobsters from each thermal treatment were euthanised in a seawater ice slurry for 5 min before haemolymph was sampled for immune and bacterial assays. Haemolymph was aspirated from the base of pereiopods and heart^23^ using a sterile ice-chilled syringe and needle. For all assays except clotting time and spread plate culture, the syringe was pre-filled with an equal volume of anticoagulant (modified from^72^; 400 mM NaCl, 0.1 M glucose, 30 mM trisodium citrate, 26 mM citric acid, 40 mM EDTA). Lobsters that died during the experiment were not sampled as haemolymph clots quickly after death rendering separation of plasma from haemocytes unfeasible for downstream analyses, but dead lobsters were counted for survival analysis (*n* = 22 for each thermal treatment).

### Immune parameters

Haemocytes including hyalinocytes (phagocytosis) and granulocytes (encapsulation, prophenoloxidase system, cytotoxicity) were measured as they play major roles in the crustacean immune response^73^. Total haemocyte counts and May-Grunwald and Giemsa staining of haemolymph smears were conducted according to Evans^23^. Large granular and small granular haemocytes were counted together as granulocytes^74^. The percentage of granulocytes was found by expressing the fraction of total granulocytes from 100 counted total haemocytes. Clotting times were analysed according to Evans^23^ and samples that did not clot before 60 s were recorded as no clot and excluded from statistical analysis.

### Culture-dependent bacterial analyses

One hundred microlitres of haemolymph from each lobster was spread-plated on Zobell marine agar (Amyl Media Pty. Ltd., Dandenong, Australia). Plates were incubated at 28 °C for up to 7 d (to allow slower growing bacteria to colonise when plates were not too crowded by overgrowth) and the number of colony forming units (CFUs) were counted. All colonies from each lobster reared at 28 °C or 34 °C on 6 dpe were isolated and cultured in Shieh broth (Amyl Media; 5 g L^−1^ bacteriological peptone, 0.1 g L^−1^ sodium pyruvate, 0.01 g L^−1^ sodium acetate, 0.5 g L^−1^ yeast extract, 0.01 g L^−1^ citric acid in 900 mL of sea water and 100 mL distilled water), pooled according to treatment group and stored in urea extraction buffer (4 M urea, 1% sodium dodecyl sulfate, 0.2 M sodium chloride, 1 mM sodium citrate; pH 8.2) prior to PCR and sequencing (Section 4.4.3).

### Culture-independent bacterial analyses

#### Sampling

One millilitre of anticoagulated haemolymph from each lobster was centrifuged at 100 *g* for 5 min to separate plasma from haemocytes. The supernatant was centrifuged at 10,000 *g* for 10 min to collect bacteria from the plasma. All but 100 μL of the supernatant was removed before adding 400 μL of urea extraction buffer and sample storage at −20 °C until further processing.

#### Total nucleic acids (TNA) extraction

Haemolymph samples were vortexed in urea extraction buffer and 100 µg of proteinase K (Bioline Pty. Ltd., NSW, Australia), heated at 55 °C for 1 h (vortexed every 5 min) and incubated on ice for 5 min. Ammonium acetate (7.5 M; Sigma-Aldrich Co., MO, USA) was added to a final concentration of 2.5 M, vortexed for 30 s and centrifuged at 14,000 *g* for 5 min (18 °C). The supernatant was mixed by inversion with 1 mL of isopropanol with 0.02 µg µL^−1^ pink co-precipitant (Bioline) and centrifuged at 16,000 *g* for 30 min. The pellet was rinsed with 500 µL of 70% ethanol twice and resuspended in 50 μL of buffered water (0.05% Triton X-100, 10 mM TRIS pH 7). Protocol modifications were made to extract TNA from pooled bacterial colonies. Each pool of colonies was put on ice for 5 min. Ammonium acetate (7.5 M) was added to a final concentration of 2.5 M, vortexed for 30 s and centrifuged at 12,000 *g* for 5 min (18 °C). The supernatant was mixed with an equal volume of isopropanol with pink co-precipitant and centrifuged at 12,000 *g* for 15 min. The pellet was rinsed with 70% ethanol twice before resuspension in 100 μL of buffered water. For purification, each TNA extract was added with an equal volume of 19% polyethylene glycol, mixed and incubated at room temperature for 15 min then centrifuged at 14,000 *g* for 15 min (18 °C). The pellet was rinsed with 200 µL of 70% ethanol, centrifuged for 14,000 *g* for 3 min and resuspended in 50 µL of buffered water.

#### PCR and pyrosequencing

A nested PCR approach was used due to the occurrence of low molecular weight non-specific PCR products when TNA extract from haemolymph was used directly in PCR with pyrosequencing primers. The primary PCR mixture consisted of 10 μL of 2 × MyTaq HS mix (Bioline), 200 nM each of 27F (5′-AGAGTTTGATCMTGGCTCAG-3′) and 1492 R (5′-GGTTACCTTGTTACGACTT-3′) 16S rRNA gene primers and 2 μL of purified TNA. The PCR was conducted using a C1000^TM^ Thermal Cycler (Bio-Rad Laboratories Inc., USA) with the following thermal cycling program: initial melting for 3 min at 95 °C; 20 cycles of denaturation for 10 s at 95 °C, annealing for 30 s at 55 °C, extension for 30 s at 72 °C; and a final extension for 3 min at 72 °C. Barcoded primers^30^ were used in the secondary PCR to amplify V1 to V3 hypervariable regions of the 16S rRNA gene. The secondary PCR contained 10 μL of 2 × MyTaq HS mix, 250 nM each of forward and reverse 16S rRNA gene primers and 2 μL of 1:10 diluted primary PCR products. The thermal cycling program for secondary PCR was initial denaturation for 3 min at 95 °C; 25 cycles of denaturation for 10 s at 95 °C, annealing for 30 s at 58 °C, extension for 10 s at 72 °C; and a final extension for 2 min at 72 °C. PCR products were examined using 1.5% agarose gel electrophoresis. All PCRs included negative extraction and amplification controls (no band on gel).

As there was no non-specific product in PCR of pooled bacterial colonies, direct PCR was used. The PCR contained 10 μL of 2 × MyTaq HS mix, 300 nM each of barcoded forward and reverse 16S rRNA gene primers and 2 μL of 1:10 diluted TNA extract. The thermal cycling program consisted of initial denaturation for 3 min at 95 °C; 30 cycles of denaturation for 10 s at 95 °C, annealing for 30 s at 58 °C, extension for 15 s at 72 °C; and a final extension for 2 min at 72 °C. PCR products were examined using 1.5% agarose gel electrophoresis.

All PCR products were concentrated using isopropanol precipitation before purification. Ammonium acetate was added to PCR products to reach a final concentration of 2.5 M. An equal volume of isopropanol with pink co-precipitant was put into the mixture, incubated on ice for 30 min and centrifuged for 16,000 *g* for 30 min. The pellet was washed twice with 70% ethanol and resuspended in buffered water. Concentrations of PCR products were determined using a Qubit^TM^ fluorometer (Invitrogen^TM^, Life Technologies Australia Pty. Ltd., VIC, Australia). Twenty five nanograms of barcoded PCR amplicon per sample (18 culture-independent and 2 culture-dependent samples were selected due to cost limitation) was pooled. The pool was purified using SureClean (Bioline) according to the manufacturer’s instructions and quantified using Qubit. A 100 µL suspension containing 2 ng µL^−1^ of PCR amplicons was sent to Macrogen Inc. (Seoul, Korea) for pyrosequencing (454 GS-FLX Titanium, Roche, USA).

#### Quantitative PCR (qPCR)

The TNA of *Yersinia ruckeri* (serotype 01b, strain UTYR001) was extracted and diluted in a ten-fold series to be used as standards. The qPCR reaction (10 uL) consisting of 5 μL of 2 × MyTaq HS mix, 400 nM each of YrF and YrR primers^75^, 100 nM of hydrolysis probe^76^ and 2 μL of *Y*. *ruckeri* TNA was processed in CFX Connect^®^ Real-Time System (Bio-Rad Laboratories Inc., USA). The qPCR program was 95 °C for 3 min and 40 cycles of 95 °C for 5 s and 60 °C for 30 s.

As the copy number of 16S rRNA gene varies between bacteria, single copy RNA polymerase beta subunit (*rpoB*) gene was used for estimating bacterial load. Two microlitres of standards and haemolymph TNA (1:2 dilution) were included in qPCR reactions (10 µL) consisting of 5 µL of 2 × MyTaq HS mix with SYBR, 400 nM each of *rpoB*1698f and *rpoB*2041r primers^77^. Duplicates of templates, standards, negative extraction and amplification controls were run in a 96 well plate. The reaction conditions were 95 °C for 3 min and 40 cycles of 95 °C for 10 s, 55 °C for 20 s and 72 °C for 10 s. Melt curve analysis was performed at 95 °C for 10 s and 58 °C for 5 s to allow reannealing before melting from 72 to 92 °C (0.5 °C increment per 5 s).

### Data analyses

For total haemocyte and granulocyte counts, and clotting time a normality test was performed before conducting either parametric (independent samples *t* test) or in the case of non-normal data, non-parametric (Mann-Whitney U test) statistic was used to compare lobsters reared at 28 °C and 34 °C at each time point. Similarly for alpha diversity indices and bacterial load, a normality test was conducted before parametric or non-parametric statistics. Data were analysed using SPSS v20. A *P* value of less than 0.05 was considered significant for all the statistical analyses.

For bacterial diversity, Geneious 8.1.7^78^ was used to demultiplex the sequence file to individual samples based on the barcodes and to trim primers. The sequences were exported to the Data Intensive Academic Grid computational cloud^79^ for use with the CloVR pipeline for 16S rRNA amplicon analysis. Chimeric and poor quality (≤100 bp, ≥2000 bp, homopolymer ≥8 bp, and ambiguous bases >0) sequences were removed by UCHIME and Qiime^80^. MOTHUR was used to cluster unique sequences, analyse richness and diversity indices and compute rarefaction curves^80^. Operational taxonomic units (OTUs) or clusters of filtered sequences with 97% nucleotide sequence identity were assigned to known bacterial taxa based in the Greengenes database v2013^81^ with a confidence threshold of 0.8 using RDP Bayesian Classifier. Good’s coverage was calculated based on the formula of (1 – [observed OTUs/number of filtered sequences]) × 100%. A biom-formatted file from CloVR was uploaded to MicrobiomeAnalyst^82^ to examine alpha diversity (observed OTUs, Chao1, ACE, Simpson, Shannon), beta diversity, core microbiome, abundance, and functional potential of OTUs in the samples. Low abundance OTUs (≤2 counts) with 10% or lower prevalence in samples were removed. Data were normalised by rarefying to the minimum library size, i.e., 2,325. The beta diversity was analysed by Bray Curtis, weighted and unweighted UniFrac distance based PCoA (OTU level) and PERMANOVA. For culture-dependent samples, actual abundance was reported as the count of each OTU was not directly proportional to the total count. MetagenomeSeq (zero-inflated Gaussian fit) compared OTU abundance (mean ± standard error) between two thermal regimes and a false discovery rate-adjusted *P* value (Q) of <0.05 was considered significant. Functional potential of OTUs was predicted using PICRUSt^69^. The functional diversity profile was generated from the sum of abundance of each OTU for each KEGG metabolism normalised by category size. The functional association analysis was used to demonstrate the KEGG pathways and *P* value of <0.05 was considered significant. A Venn diagram showing numbers of shared and unique OTUs among samples was generated using InteractiVenn^83^.

For bacterial load using qPCR, target amplicons were distinguished from non-specific amplification of primer dimers by melt curve analysis. The target amplicon was not present in negative extraction and amplification controls. The number of 16S rRNA copies in the *Y*. *ruckeri* standards were determined using CM3 model^76^ in the qPCR package of RStudio (v3.2.3) software. The number of 16S rRNA copies in the standards were converted to the cell equivalents to allow construction of a standard curve for the samples (*rpoB* gene).

## Supplementary information

Supplementary Fig. 1.

## Data Availability

The sequences generated during the current study are available in the NCBI Sequence Read Archive under BioProject accession PRJNA445187.
