# Supplementary material for: Haemolymph microbiome of the cultured spiny lobster Panulirus ornatus at different temperatures
Source: Sci Rep. 2019 Feb 8;9:1677. doi: 10.1038/s41598-019-39149-7 (PMC6368590; doi:10.1038/s41598-019-39149-7)
Supplement: Supplementary file 1 — Supplementary Fig. 1. [file 41598_2019_39149_MOESM1_ESM.pdf]

Haemolymph microbiome of the cultured spiny lobster *Panulirus ornatus* at different temperatures

Mei C. Ooi, Evan F. Goulden, Gregory G. Smith, Andrew R. Bridle

Supplementary information

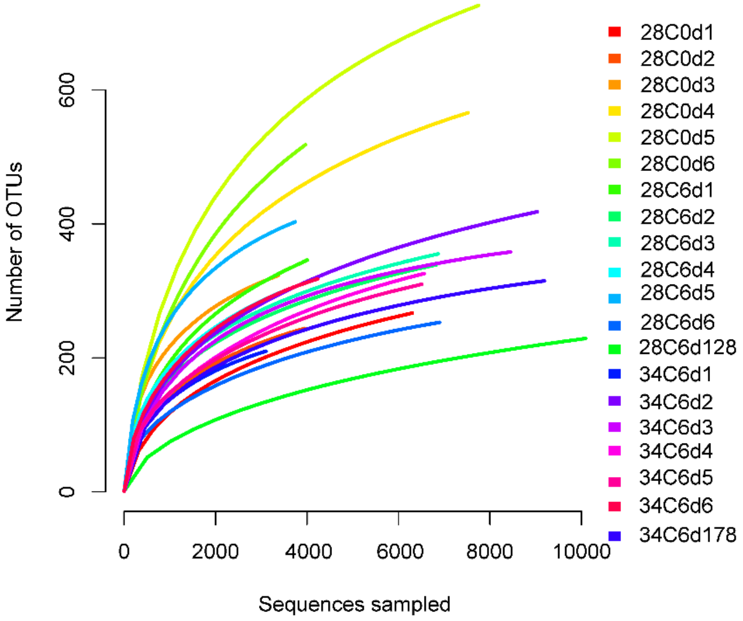

Supplementary Fig. 1. Rarefaction curves for haemolymph sequence libraries of *P. ornatus* juveniles (see Table 1 for abbreviation of samples).
